# Organization of the Mammalian Locomotor CPG: Review of Computational Model and Circuit Architectures Based on Genetically Identified Spinal Interneurons

**DOI:** 10.1523/ENEURO.0069-15.2015

**Published:** 2015-09-22

**Authors:** Ilya A. Rybak, Kimberly J. Dougherty, Natalia A. Shevtsova

**Affiliations:** Department of Neurobiology and Anatomy, Drexel University College of Medicine, Philadelphia, Pennsylvania 19129

**Keywords:** central pattern generator, computational modeling, flexor–extensor coordination, genetically identified neurons, left–right coordination, locomotion, spinal cord

## Abstract

The organization of neural circuits that form the locomotor central pattern generator (CPG) and provide flexor–extensor and left–right coordination of neuronal activity remains largely unknown. However, significant progress has been made in the molecular/genetic identification of several types of spinal interneurons, including V0 (V0_D_ and V0_V_ subtypes), V1, V2a, V2b, V3, and Shox2, among others. The possible functional roles of these interneurons can be suggested from changes in the locomotor pattern generated in mutant mice lacking particular neuron types. Computational modeling of spinal circuits may complement these studies by bringing together data from different experimental studies and proposing the possible connectivity of these interneurons that may define rhythm generation, flexor–extensor interactions on each side of the cord, and commissural interactions between left and right circuits. This review focuses on the analysis of potential architectures of spinal circuits that can reproduce recent results and suggest common explanations for a series of experimental data on genetically identified spinal interneurons, including the consequences of their genetic ablation, and provides important insights into the organization of the spinal CPG and neural control of locomotion.

## Visual Overview

**Figure:**
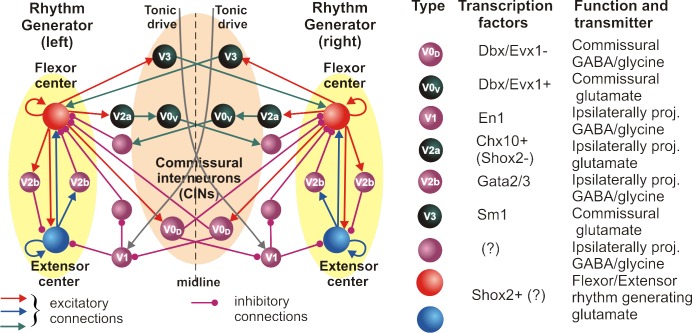

## Significance Statement

We review and analyze together (1) the currently proposed architectures of the central pattern generator (CPG) in the mammalian spinal cord and (2) different classes of genetically identified spinal interneurons. We suggest the possible roles of these interneurons, and their connectivity and functions within the spinal circuitry and the CPG, which can simultaneously explain multiple experimental data and provide important insights into the organization and operation of neural circuits in the spinal cord during locomotion.

## Introduction

The nervous systems of vertebrates contain neural networks representing central pattern generators (CPGs) that generate neural oscillations and control rhythmic movements. These CPGs are involved in many important functions, including different forms of locomotion, such as swimming, walking, running, and flying. Interplay between modeling and experimental studies has been valuable in advancing our understandings of different vertebrate CPGs (Lansner et al., 1998; Ijspeert, 2001; Grillner, 2006; Rybak et al., 2006a,b; Grillner et al., 2007; McCrea and Rybak, 2008; Wolf et al., 2009; Zhong et al., 2012; for review, see Rybak, 2014). Computational modeling provides powerful tools through which experimental findings and current hypotheses from different laboratories can be incorporated together in a common computational framework allowing the generation of plausible predictions for future experimental studies. In this review, we focus on the mammalian locomotor CPG, and merge recent experimental and computational findings to produce a more complete common framework.

The vertebrate locomotor CPGs are located in the spinal cord. The first schematic of the locomotor CPG, called the “half-center” model, was proposed by Graham Brown (1914) and elaborated on by Lundberg (1981; for review, see McCrea and Rybak, 2008; Stuart and Hultborn, 2008). This model included two (flexor and extensor) half-centers reciprocally inhibiting each other. The mutual inhibitory interactions between the half-centers were mediated by inhibitory interneurons that ensured that only one half-center could be active at a time. The activity of the active half-center gradually reduced due to some fatigue or adaptation mechanism, leading to the activation of the antagonistic half-center. The antagonistic half-center then inhibited the active half-center, hence switching the locomotor phase. It was suggested that the flexor and extensor half-centers directly project to and activate the flexor and extensor motoneurons, respectively. Studies on the immobilized decerebrate cat demonstrated that continuous electrical stimulation of the midbrain locomotor region (MLR) can produce “fictive locomotion”—the rhythmic pattern of motoneuron activity that is characterized by alternating activation of flexor and extensor motoneurons similar to that observed during normal locomotion in the intact animal (Rossignol, 1996; Markin et al., 2012). A similar pattern of locomotor activity can also be produced by systemic administration of the noradrenergic precursor, l-DOPA (Jankowska et al., 1967a,b). The demonstration that both MLR stimulation and neuroactive drug application can evoke fictive locomotion provided strong evidence for the existence of locomotor CPG in the mammalian spinal cord.

In contrast to many other neural networks in the brain, whose architecture, neuron types, and connectivity have been mainly or partly determined, the spinal cord circuitry does not exhibit a clear spatial organization, which makes it difficult to distinguish network elements with specific functions using traditional electrophysiological methods in order to reconstruct and suggest a realistic circuit organization from these data. Significant progress in this direction has been recently achieved due to the identification of several types of spinal interneurons during development based on the expression of transcription factors and the use of isolated spinal cord preparations from genetically modified mice lacking particular neuron types (Jessell, 2000; Lanuza et al., 2004; Gosgnach et al., 2006; Kiehn, 2006, 2011; Zhang et al., 2008, 2014; Goulding, 2009; Gosgnach, 2011; Kiehn, 2011; Kiehn and Dougherty, 2013; Talpalar et al., 2013).

To date, a series of spinal interneurons has been genetically identified and functionally characterized using these approaches. At the same time, the exact functional roles of these neurons remain purely understood, and their possible location, connectivity, and functions within the currently considered computational architectures of spinal and CPG circuits have not been considered and analyzed so far. In this review, we have made an attempt to include these neurons, and their potential interactions and functions, in a common logical and computational framework to propose explanations for multiple experimental data and provide important insights into the organization and operation of neural circuits in the spinal cord during locomotion.

## Models of the locomotor CPG

**Figure 1.**
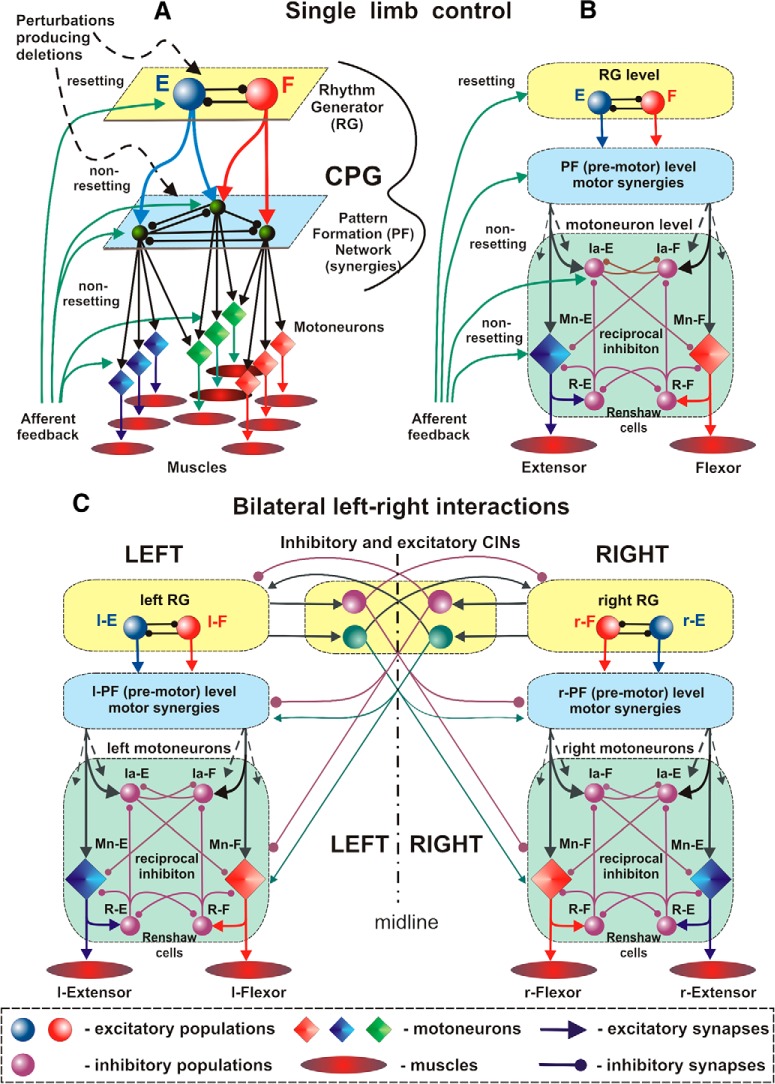
Two-level model of locomotor CPG and left–right commissural interactions. ***A***, A two-level functional organization is suggested to include bipartite half-center RG and PF circuits that mediate RG control of motoneuron activity and distribute RG inputs to functionally synergist motoneuron pools. Different perturbations and afferent stimuli affecting the system at the RG level may reset the rhythm (produce resetting deletions), whereas perturbations and afferent signals acting at the PF or motoneuron level cannot reset the rhythm and can only produce non-resetting deletions. ***B***, Organization of interactions at the lower, motoneuron level, including flexor and extensor motoneurons (Mn), Ia interneurons, and Renshaw cells (R). ***C***, Organization of bilateral left–right interactions in the spinal cord mediated by inhibitory and excitatory CINs. E, Extensor; F, flexor; l, left; r, right.

The classical half-center concept initially proposed by Graham Brown and Lundberg  represents only a general, simplified CPG organization and cannot reproduce and explain many features of the real locomotor pattern generated in the mammalian spinal cord. The actual locomotor pattern does not exhibit strictly alternating flexor and extensor activities with all motoneurons clearly belonging to one of these two groups. The real pattern is more complicated and includes motoneuron pools (e.g., the ones controlling biarticular muscles) that exhibit bursts in each locomotor phase or only a very short burst within one phase. There are also noticeable differences in the timing of burst onset and/or offset between different flexor and/or between different extensor motor pools. To overcome these and other limitations of the classical half-center model, Grillner (1981) proposed a unit burst generator (UBG) concept of CPG organization that suggested the existence of several separate rhythmogenic modules (or unit burst generators) controlling each joint of the limb and interacting with each other as coupled neural oscillators. The other solution, proposed by Rybak et al. (2006a,b) and McCrea and Rybak (2007, 2008), suggested that the spinal CPG has a two-level functional organization consisting of a bipartite half-center rhythm generator (RG) and pattern formation (PF) circuits (Fig. 1*A*,*B*). In this organization, the PF circuits mediate RG control of motoneuron activity and distribute RG activity to functionally distinct populations of interneurons projecting to groups of synergist motoneuron pools. The motoneuron pools controlled by a common source at the PF level display a synchronized activity and represent motor synergies controlled by the CPG. During locomotion, these synergies may be refined by both descending signals from higher centers and peripheral afferent feedback. The potential advantages and disadvantages, and the plausibility of the UBG and two-level concepts are still debated in the literature (McCrea and Rybak, 2008; Stuart and Hultborn, 2008; Zhong et al., 2012; Hägglund et al., 2013; Wiggin et al., 2012; Grillner and Manira, 2015; McLean and Dougherty, 2015). Independent of CPG organization, there is also a lower motoneuron level in the spinal cord that contains multiple circuits of local reflexes defined by special interactions between the populations of particular motoneurons, Renshaw cells, and interneurons involved in nonreciprocal and reciprocal antagonist interactions and processing of afferent feedback (Ia, Ib, and other types; Fig. 1*B*). In the framework of a multilevel CPG, any spontaneous perturbation or an afferent or supraspinal signal affecting spinal circuitry below the RG level (i.e., at the PF or motoneuron levels) may produce a non-resetting deletion (i.e., the omission of one or more bursts in the output motor activity) or may cause a delay or earlier onset of the next burst without a general phase shift in the post-perturbation or post-deletion rhythm relative to the pre-perturbation or pre-deletion rhythm (i.e., without rhythm resetting). Resetting perturbations and deletions can only happen at the RG level (Rybak et al., 2006a,b; McCrea and Rybak, 2007, 2008; Zhong et al., 2012; Fig. 1*A*,*B*). Therefore, the analysis of burst deletions in the activity of different spinal neurons may suggest the level of their operation.

 It is generally accepted that in limbed vertebrates, including mammals, there is a separate CPG controlling each limb, whose rhythmic movements are coordinated with the movements of other limbs via neural pathways within the cord and supraspinal and afferent signals (Grillner, 1981, 2006; Orlovsky et al., 1999; McCrea and Rybak, 2008). Specifically, the left–right limb coordination is mediated by inhibitory and excitatory commissural interneurons (CINs), whose axons cross the midline and affect neurons located in the contralateral side of the cord (Kjaerulff and Kiehn, 1997; Butt and Kiehn, 2003; Kiehn and Butt, 2003; Lanuza et al., 2004; Quinlan and Kiehn, 2007; Jankowska, 2008; Kiehn, 2011; Talpalar et al., 2013). The identified CINs were shown to provide inhibitory and excitatory synaptic inputs to contralateral motoneurons (Butt and Kiehn, 2003; Kiehn and Butt, 2003; Quinlan and Kiehn, 2007). However, the effects of synaptic inputs from the CINs to contralateral motoneurons are supposed to be weaker than the effects of inputs that these motoneurons receive from the ipsilateral CPG, so that under normal conditions the contralateral signals do not override the ipsilateral drives to the corresponding motoneurons. To coordinate left and right rhythmic activities (e.g., alternate or synchronize them), CINs should not only project to contralateral motoneurons, but should primarily mediate mutual interactions between the left and right rhythm generators (Fig. 1*C*). The effects of the CINs on either RG interneurons or motoneurons cannot be significant since the genetic ablation or silencing of CINs (e.g., V0 and V3 types) did not exhibit obvious changes in the frequency or amplitude of locomotor activity recorded from ventral roots, even when left–right alternation of activity was changed to left–right synchrony (Lanuza et al., 2004; Zhang et al., 2008; Talpalar et al., 2013). This means that, although left–right interactions mediated by inhibitory and excitatory CINs do not affect the amplitude and frequency of locomotor oscillations, they are sufficient to coordinate phase relationships between rhythmic activities generated by left and right RGs, and hence to define the gait of locomotion.

## Spinal interneurons identified from developmental and genetic studies

**Figure 2.**
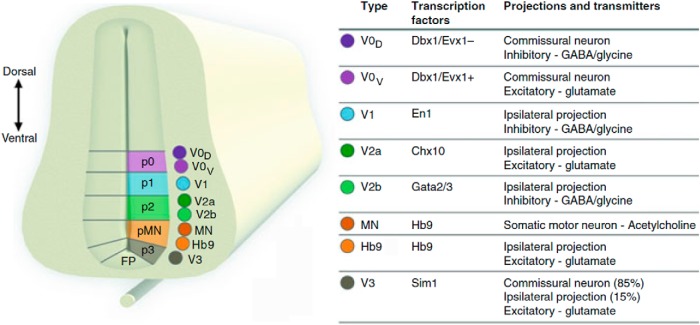
Molecular code determines the identity of ventral spinal neurons. Morphogens secreted from the floor plate and roof plate set up concentration gradients in the ventricular zone to specify progenitor domains p0–p3 and pMN, characterized by their differential expression of transcription factors. When the progenitor cells mature, they migrate laterally and are called V0–V3, Hb9, and motor neurons. The table on the right depicts the main transcription factors in the five cardinal classes of ventrally located neurons (V0–V3, Hb9) and motor neurons, the projection pattern, and the transmitter phenotype of these neurons. Dbx1, developing brain homeobox 1; Evx1, even-skipped homeobox; En1, engrailed 1; Chx10, Ceh-10 homeodomain-containing homolog; Gata2/3, GATA binding proteins 2 and 3; Sim 1, single-minded homolog 1; Hb9, homeobox 9; FP, floor plate. Reproduced from Kiehn and Dougherty (2013), their Figure 38.8, with permission.

 Over the past decade, a novel experimental approach incorporating genetic and molecular techniques enabled the determination of key neural elements of the spinal circuits and locomotor CPG (Jessell, 2000; Goulding, 2009). Molecular genetic experiments have shown that the developing neural tube in the embryonic mouse can be divided into distinct populations of spinal neurons based on the expression of transcription factors. Specifically, transcription factors were used to identify several classes of ventral horn interneurons, including V0–V3 types, some of which were further divided into subtypes (Fig. 2; for review, see Goulding, 2009; Gosgnach, 2011; Kiehn, 2011; Kiehn and Dougherty, 2013).

The V0 neurons settle in the ventromedial spinal cord. They can be identified by the expression of the transcription factor Dbx1 (Pierani et al., 2001; Lanuza et al., 2004; Talpalar et al., 2013). The subdivision of V0 neurons into subtypes is based on transmitter phenotype, and these subtypes include dorsally located inhibitory V0_D_ neurons (Pax7, Evx1^−^), ventrally located glutamatergic V0_V_ neurons (Evx1), and cholinergic V0_C_ neurons (Pitx2) located near the central canal. Both V0_D_ and V0_V_ subtypes project their axons contralaterally (Pierani et al., 2001) and represent major types of CINs involved in left–right alternation of neuronal activity in the spinal cord (Lanuza et al., 2004; Talpalar et al., 2013). Moreover, the selective genetic ablation of these neurons has shown that these types of CINs are involved in left–right alternation in a speed-dependent manner (Talpalar et al., 2013). The V0_D_ neurons are essential for left–right alternation at slow locomotor speeds, and their ablation has little effect on locomotion at higher speeds. In contrast, the V0_V_ ablated mice show normal left–right alternation at slow speeds but switch to left–right synchronous activity at higher locomotor speeds (Talpalar et al., 2013).

The V1 neurons, expressing En1, are a heterogeneous group of inhibitory, ipsilaterally projecting interneurons (Fig. 2). Different subtypes of these neurons are involved in different inhibitory functions in the spinal cord, such as reciprocal (Ia interneurons) and recurrent (Renshaw cells) inhibition. A subpopulation of these neurons was found necessary for generating fast locomotor activity and, hence, can play a role in regulating the locomotor speed (Gosgnach et al., 2006; Zhang et al., 2014). These neurons, along with V2b neurons (see below), are involved in securing flexor–extensor alternation in the spinal cord. Abrogating neurotransmission in both V1 and V2b neuron types in the isolated spinal cord resulted in a synchronous flexor and extensor locomotor activity (Goulding et al., 2014; Zhang et al., 2014).

The V2 neurons express Lhx3 and are all ipsilaterally projecting. Two major subtypes of V2 neurons include (Fig. 2): excitatory V2a, expressing Chx10; and inhibitory V2b, expressing Gata2 or Gata3 (Al-Mosawie et al., 2007; Lundfald et al., 2007). During drug-induced fictive locomotion in isolated spinal cord, about half of the V2a interneurons receive rhythmic synaptic drive, which increases with locomotor frequency, recruiting additional V2a neurons at higher locomotor frequencies (Zhong et al., 2011). Genetic ablation of V2a neurons results in changes similar to those produced by the ablation of V0_V_ neurons, as follows: mutant mice maintain left–right alternating activity at low locomotor speeds and switch to left–right synchrony at high speeds (Crone et al., 2008, 2009). The similarity in the locomotor patterns seen following V2a and V0_V_ neuronal ablations suggests that a subpopulation of V2a neurons provides excitatory drive to the V0_V_ commissural pathway (Kiehn, 2011; Shevtsova et al., 2015). This is further supported by the demonstration of V2a terminals on V0_V_ somata (Crone et al., 2008).

As mentioned above, the V2b neurons, along with V1 neurons, support flexor–extensor alternation in the intact cord, which can be disturbed only by silencing both of these neuron types (Zhang et al., 2014; Goulding et al., 2014). Flexor–extensor alternation is also maintained in a hemisected spinal cord, where this alternation is entirely supported by V2b neurons. Synaptic silencing of V2b neurons in hemicords results in flexor–extensor synchrony (Zhang et al., 2014). Therefore, in contrast to V1 neurons, the activity of V2b neurons depends on inputs from ipsilateral circuits.

The V3 neurons express Sim1 and are mainly excitatory commissural neurons (Zhang et al., 2008). Silencing of these neurons does not affect left–right or flexor–extensor alternation. It is suggested that V3 neurons participate in maintaining a stable, symmetrical rhythm; silencing of these neurons increases the variability of bursting amplitude and durations (Zhang et al., 2008).

## Spinal interneurons involved in locomotor rhythm generation: Shox2 cells

**Figure 3.**
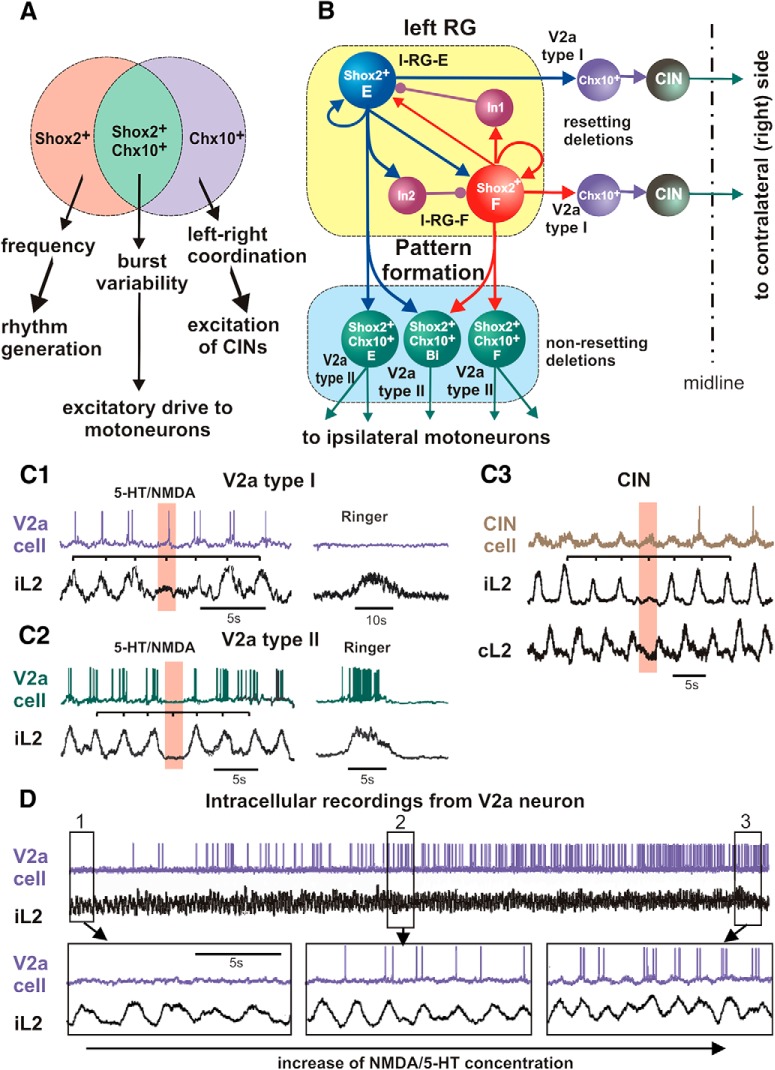
Shox2^+^ neurons and two types of V2a (Chx10^+^) cells. ***A***, The suggested different functional roles of Shox2^+^ (non-V2a), Shox2^+^ Chx10^+^ (V2a), and Shox2^off^ Chx10^+^ (V2a) neurons. Reproduced from Dougherty et al. (2013), their Figure 8A, with permission. ***B***, Different roles of V2a type I (Chx10^+^ Shox2^off^) and V2a type II (Chx10^+^ Shox2^+^) neurons. ***C1***, V2a type I neuron continued to receive rhythmic excitatory synaptic inputs and fired rhythmic bursts during a non-resetting ipsilateral flexor deletion (iL2) occurring during NMDA/5-HT-induced fictive locomotion (current-clamp recording). Deletion is indicated by the pink bar. Recordings on the right show that this neuron did not receive any synaptic drive during a spontaneous nonlocomotor activity observed in the ipsilateral motor output (iL2). ***C2***, V2a type II neuron was silent and lost synaptic drive during a non-resetting flexor (iL2) deletion. This neuron was excited and fired a prolonged burst of action potentials during a spontaneous nonlocomotor iL2 burst of activity (right). ***C3***, Activity of a flexor-related CIN during a non-resetting flexor deletion. One can see rhythmic membrane potential oscillations in phase with the iL2 root activity, and the neuron continued to oscillate during flexor deletion. ***C1***, ***C2***, and ***C3*** are reproduced from Zhong et al. (2012), their Figures 5D,E, 6C,D, and 4B, respectively, with permission. ***D***, Intracellular recording from V2a neuron exhibiting an increased firing activity with increased drug (NMDA/5-HT) concentration. Insets 1, 2, and 3 show expanded recordings indicated in the top diagram. The iL2 trace represents smoothed and filtered ventral root activity. Reproduced from Zhong et al. (2011), their Figure 4a*–*d, with permission.

 The excitatory, ipsilaterally projecting V2a neurons could be considered potential candidates for the rhythm-generating neurons in the mammalian spinal cord. However, ablation of these neurons does not affect the rhythm (Crone et al., 2008). Difficulties in the identification of specific rhythm-generating neurons may occur because these neurons come from dorsal progenitors that migrate ventrally or because they are spread sparsely across known ventral neuron classes. Shox2 expression was found in a subset of Chx10-expressing V2a neurons, and in non-V2a neurons coming from various dorsal and ventral progenitors (Fig. 3*A*). All of these neurons are excitatory and ipsilaterally projecting (Dougherty et al., 2013). Most Shox2^+^ neurons are rhythmically active during locomotion, and they show heterogeneity in target projections, projecting to other Shox2^+^ neurons, CINs, and/or motoneurons (Fig. 3*A*). Either short-term or long-term silencing of the Shox2 population resulted in a reduction in the locomotor frequency measured from ventral root recordings during both drug-evoked and brainstem stimulation-evoked locomotion, suggesting that they play a role in rhythm generation. Ablation of the Shox2^+^ Chx10^+^ (V2a) subset resulted in an increase in the variability of burst amplitude and cycle period but had no effect on locomotor frequency. Therefore, only the Shox2^+^ Chx10^−^ (non-V2a) neurons were concluded to be part of the rhythm generator for locomotion (Fig. 3*A*). However, a rhythm remained, albeit slower, after Shox2 silencing, demonstrating that Shox2^+^ neurons are not the only rhythm-generating neurons. Other components of the rhythm generator are not known at this time. The only current candidate is the Hb9 interneuron population (Fig. 2), which is also suggested to be involved in rhythm generation (Wilson et al., 2005; Hinckley and Ziskind-Conhaim, 2006; Brocard et al., 2010, 2013). It was shown that their activity does not precede burst initiation in motor outputs, suggesting that they may contribute to maintenance of the locomotor rhythm but cannot represent the sole rhythm-generating kernel (Kwan et al., 2009). Whether they are another constituent population of the rhythm generator remains to be directly tested.

The intrinsic neuronal properties involved in generating rhythmic bursting in Shox2 cells are currently unknown. A series of previous models of the mammalian spinal rhythm generator suggested that this rhythmic activity is based on the persistent sodium current, *I*_NaP_ (Rybak et al., 2006a,b,2014; McCrea and Rybak, 2007; Sherwood et al., 2011; Zhong et al., 2012; Brocard et al., 2013). This suggestion was indirectly supported by a series of experimental studies performed in different laboratories (Zhong et al., 2007; Tazerart et al., 2007, 2008; Ziskind-Conhaim et al., 2008; Brocard et al., 2010, 2013). Moreover, the presence and involvement of *I*_NaP_ for rhythm generation in spinal Hb9 neurons has been explicitly demonstrated (Brocard et al., 2013).

## Ipsilaterally projecting, excitatory V2a interneurons

 Based on Shox2 expression, the population of V2a (Chx10) neurons can be divided into the following two groups: Shox2^+^ and Shox2^−^ (Fig. 3*A*; Dougherty et al., 2013). When Shox2-expressing V2a neurons were ablated, there was no effect on left–right coordination. The only observable phenotype was an increase in the variability of burst amplitude and cycle period, which was also seen in the complete Shox2 mutants and the V2a mutants. This, together with the study by Crone et al. (2008, 2009), indicates that the V2a neurons that do not express Shox2 (Chx10^+^ and Shox2^−^) are the neurons responsible for left–right deficits seen in the V2a ablated mice, and the Shox2^+^ V2a neurons provide input to motoneurons (Fig. 3*A*,*B*).

The functional separation of Shox2^+^ Chx10^+^ and Shox2^−^ Chx10^+^ neurons fits very well with the results of a previous study, Zhong et al. (2012), which also identified two different subtypes of V2a interneurons. In that study, the neurons were classified based on analysis of their activity during non-resetting deletions. According to that classification, the V2a type I neurons maintained rhythmic activity at the times when bursts in the motor output (ipsilateral ventral root) were missed (Fig. 3*C1*), whereas the V2a type II neurons had missing activation consistent with the burst deletions seen in the motor output (Fig. 3*C2*). In addition, during nonlocomoting conditions (NMDA and 5-HT washed out), the V2a type I neurons did not show activation together with ventral root “nonlocomotor” bursts (Fig. 3*C1*), whereas the V2a type II neurons did exhibit activation consistent with such nonlocomotor activity in the motor output (Fig. 3*C2*). Interestingly, the commissural neurons identified in this study also maintained bursts during non-resetting deletions in the motor output (Fig. 3*C3*), similar to V2a type I. All of these data are consistent with the different roles of V2a type I (Chx10^+^ Shox2^−^) neurons and V2a type II (Chx10^+^ Shox2^+^) neurons in the spinal circuitry suggested above. The former neurons project to CINs and contribute to left–right alternation, whereas the latter neurons may belong to PF and mediate RG input to ipsilateral motoneurons (Fig. 3*A*,*B*).

There are some other important properties of V2a neurons. Some of these neurons show a strong increase in activity and recruitment as NMDA/5-HT concentrations, and therefore locomotor frequency, are increased (Zhong et al., 2011; Fig. 3*D*). Additionally, genetic ablation of these neurons disturbed left–right alternation at high locomotor frequencies (Crone et al., 2008, 2009), suggesting that these neurons play a critical role in the support of left–right alternation in the spinal cord at high locomotor frequencies.

## Flexor–extensor asymmetry in the organization of locomotor activity

**Figure 4.**
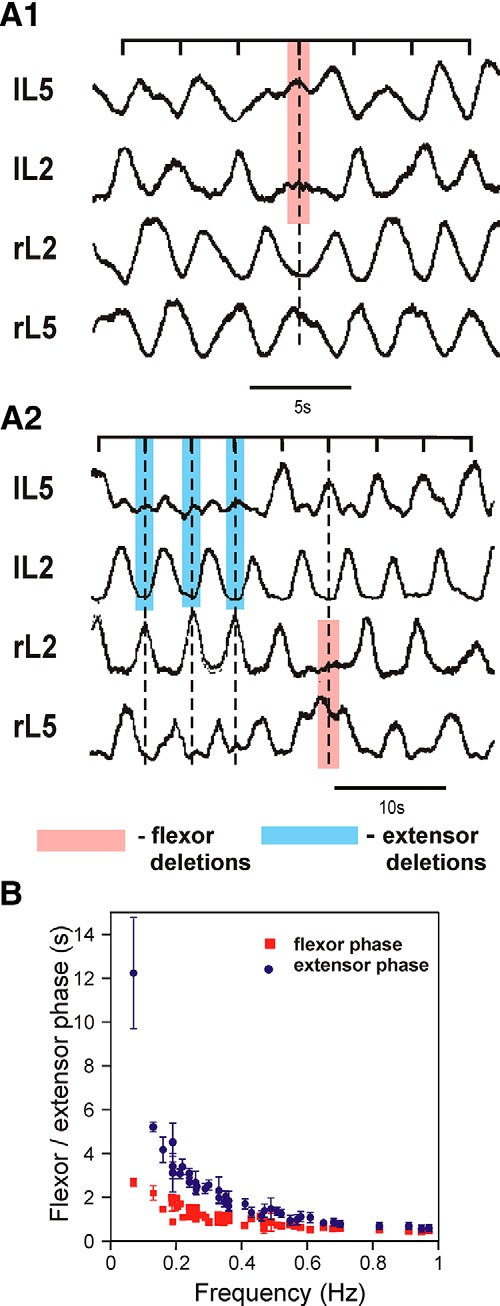
Flexor–extensor asymmetry. ***A1***, ***A2***, Flexor and extensor deletions during NMDA/5-HT-induced fictive locomotion in the isolated rat spinal cord. Smoothed and rectified traces of motoneuron activity recorded from the left (lL2, lL5) and right (rL2, rL5) lumbar ventral roots. L2 recordings show predominantly flexor motoneuron activity, while L5 recordings show predominantly extensor activity. The bars above each set of traces show the expected timing of the bursts if the rhythm were unperturbed during the deletion. ***A1*** shows an example of a non-resetting flexor deletion recorded from the lL2 root (indicated by the pink bar) accompanied by tonic activity in the ipsilateral extensor (lL5) root, but with no obvious effects on flexor or extensor activity on the opposite side of the cord. ***A2*** shows non-resetting extensor deletions (marked by blue bars) and a flexor deletion (marked by pink bar). During extensor deletions in lL5, the ipsilateral flexor activity (lL2) and contralateral activities were not perturbed, while the flexor deletion in rL2 was accompanied by tonic activity in the rL5 ventral root. Reproduced from Zhong et al. (2012), their Figure 1Aa,Ba, with permission. ***B***, Changes in flexor and extensor phase durations during NMDA/5-HT-induced fictive locomotion in the isolated mouse spinal cord as a function of locomotor frequency. Red filled squares and blue filled circles show average flexor and extensor phase durations, respectively. Reproduced from Shevtsova et al. (2015), their Figure 9C, with permission.

 Most bipartite models, from the first model of Graham Brown (1914) to the two-level model of McCrea and Rybak (2008), assumed a symmetrical organization of the rhythm generators in regard to flexor and extensor half-centers and their interactions. Nevertheless, asymmetric, flexor-dominated architectures of rhythm generation have been also proposed (Pearson and Duysens, 1976; Duysens, 1977; Zhong et al., 2012; Duysens et al., 2013; Machado et al., 2015). Two recent findings from studies in the isolated rodent spinal cord with drug-induced fictive locomotion have recently provided indirect support of the asymmetric flexor-dominated organization. First, the analysis of non-resetting deletions in these preparations revealed only the following two types of deletions: missing bursts in the flexor-dominated ventral root (L2) were accompanied by sustained activity in the ipsilateral extensor-dominated root (L5); whereas missing extensor bursts usually occurred without obvious disturbances of the ipsilateral flexor rhythmic activity (Zhong et al., 2012; Fig. 4*A*). Second, the analysis of changes in the duration of flexor and extensor phases with an increase in oscillation frequency during NMDA/5-HT-evoked fictive locomotion in the isolated mouse spinal cord has shown that the duration of flexor phase changes much less than the duration of the extensor phase (Talpalar et al., 2013; Shevtsova et al., 2015; Fig. 4*B*), which also fits with similar phase measurements performed in cats *in vivo* (Halbertsma, 1983; Frigon and Gossard, 2009). Therefore, one can suggest that extensor-related neurons in the spinal rhythm generator do not generate intrinsic rhythmic activity themselves but exhibit rhythmic bursting due to inhibition from the corresponding flexor-related rhythm-generating neurons. In this case, extensor phases may represent intervals of tonic activity between inhibitory states corresponding to flexor bursts. The potential dominance and evolutionary primacy of flexor activity have been confirmed recently by Machado et al. (2015).

The above suggestion, however, looks contradictory to multiple data showing that extensor rhythmic activity can, under certain conditions, be generated without flexor activity. Specifically, using an optogenetic approach, Hägglund et al. (2013) have demonstrated that a locomotor-like rhythmic bursting can be induced unilaterally and independently in flexor and extensor networks. A resolution of this contradiction could be achieved based on a suggestion that both flexor-related and extensor-related centers are intrinsically rhythmogenic, but the expression of their intrinsic rhythmicity may depend on various factors, such as general neuronal excitability or external drive to neurons in these populations (Shevtsova et al., 2015; or Molkov et al., 2015). The other important suggestion here is that, during locomotor activity, intrinsic rhythmicity is always present in flexor RG circuits, but the rhythmicity of extensor RG circuits is conditional and may depend on, for example, the type of experimental preparations (various *in vivo* preparations, isolated spinal cord *in vitro*) or the methods used to evoke the rhythm (neuroactive drug application, electrical stimulation of brainstem, afferent/dorsal root stimulation; Shevtsova et al., 2015).

This idea was explicitly implemented in the models of Shevtsova et al. (2015) and Molkov et al (2015), in which both flexor and extensor rhythm-generating populations (representing the corresponding locomotor centers or half-centers) could generate rhythmic bursting depending on conditions, but normally only flexor populations (centers) on each side operated in the intrinsic bursting regime while the extensor populations operated in a state of tonic activity and exhibited bursting due to the rhythmic inhibition from the ipsilateral flexor centers.

**Figure 5.**
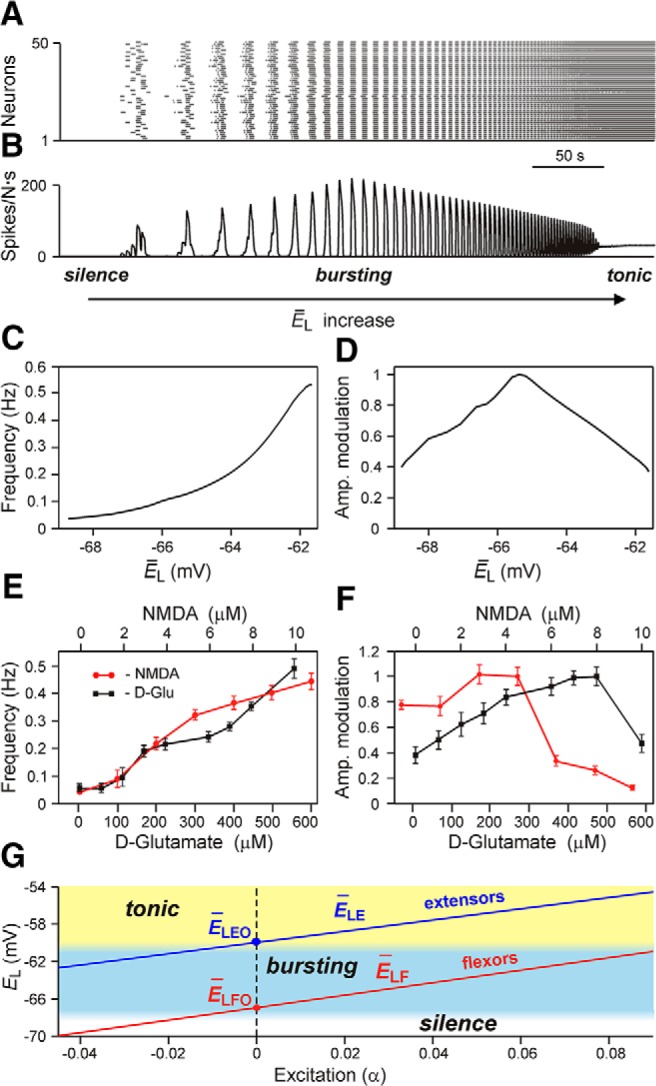
Modeling of an isolated rhythm-generating population. ***A***, Raster plot of the activity of 50 neurons from the 200-neuron population. Each horizontal line represents a neuron, and each dot represents a spike. ***B***, Integrated population activity represented by the average histogram of population activity [spikes/(neuron × s), bin = 100 ms]. In ***A*** and ***B***, the average leakage reversal potential (*Ē*_L_) defining the average level of neuronal excitation was linearly increased from −70 to −58 mV for 400 s. Voltage regions of silence, bursting, and tonic activity are denoted at the bottom. Bursting emerges at lower values of *Ē*_L_ in a limited number of neurons. With increasing *Ē*_L_, more neurons become involved and the population bursting becomes strongly synchronized. A further increase of *Ē*_L_ leads to a transition to tonic activity. ***C*** and ***D***, respectively, show the frequency of and amplitude of population activity as functions of *Ē*_L_. ***E*** and ***F***, respectively, show the frequency and amplitude of NMDA/5-HT-evoked locomotor activity in the isolated mouse spinal cord recorded *in vitro* from the flexor ventral root as a function of NMDA (red circles) or d-glutamate (black squares) concentration (the amplitude was normalized with respect to the maximal amplitude). Graphs display the mean ± SD (*n* = 20 each). ***G*** shows changes of *Ē*_L_ for flexor (*Ē*_LF_, red line) and extensor (*Ē*_LE_, blue line) RG centers during increasing neuronal excitation with an increase in drug concentration [defined by parameter α, (*Ē*_Li_ = *Ē*_LiO_ · (1 − α))] across areas for silence (white), bursting (blue), and tonic (yellow) population activity. This figure is reproduced from Shevtsova et al. (2015), their Figure 2, with permission.

Each RG population in the model of Shevtsova et al. (2015), representing either flexor or extensor centers, consisted of 200 neurons modeled on the Hodgkin–Huxley style. The intrinsic rhythmic bursting in each population was based on a persistent (slowly inactivating) sodium current, *I*_NaP_, incorporated into each neuron, and sparse mutual excitatory synaptic interactions within the population. Figure 5*A–D* shows the results of modeling a single isolated RG population during a slow ramp increase of neuronal excitation, defined by the average neuronal leakage reversal potential *Ē*_L_. At low values of *Ē*_L_, the population was silent (Fig. 5*A*,*B*). Bursting emerged as *Ē*_L_ depolarized (Fig. 5*A*,*B*; see also Fig. 5*G*, blue area). With a further increase of *Ē*_L_, the burst frequency monotonically increased (Fig. 5*B*,*C*). The burst amplitude initially went up (Fig. 5*B*,*D*), because of the increasing synchronization of bursts generated by individual neurons. Then, with further depolarization, the amplitude progressively decreased (Fig. 5*B*,*D*) due to *I*_NaP_ inactivation in each neuron, leading to a reduction of spike frequency in neuronal bursts (Butera et al., 1999; Rybak et al., 2004). Finally, for the larger (most depolarized) values of *Ē*_L_, bursting switched into sustained or tonic activity (Fig. 5*A*,*B*; see also Fig. 5*G*, yellow area). A similar transition from a silent state to bursting and tonic spiking with a progressive increase of *Ē*_L_ was shown in models of other neural oscillators (Cymbalyuk et al., 2002). The simulated effects of the increase of neuronal excitation on the population burst frequency and amplitude (Fig. 5*C*,*D*) are qualitatively similar to the reported effects of increased concentrations of neuroactive drugs, such as NMDA, on the frequency and amplitude of locomotor oscillations in the isolated spinal cord (Talpalar and Kiehn, 2010). In these experiments, the bursting frequency recorded from the flexor ventral root (L2) increased with increased drug concentration, while the amplitude initially increased and then decreased (Fig. 5*E*,*F*). This correspondence between the experimental and modeling data allowed the suggestion that the experimentally observed increase of locomotor frequency with increasing NMDA concentration resulted from a drug-induced increase in the average neuronal excitability defined in the model by *Ē*_L_. Therefore, changes in were used in the model as a tool for changing locomotor frequency to fit different speeds of drug-evoked locomotion (Shevtsova et al. 2015).

**Figure 6.**
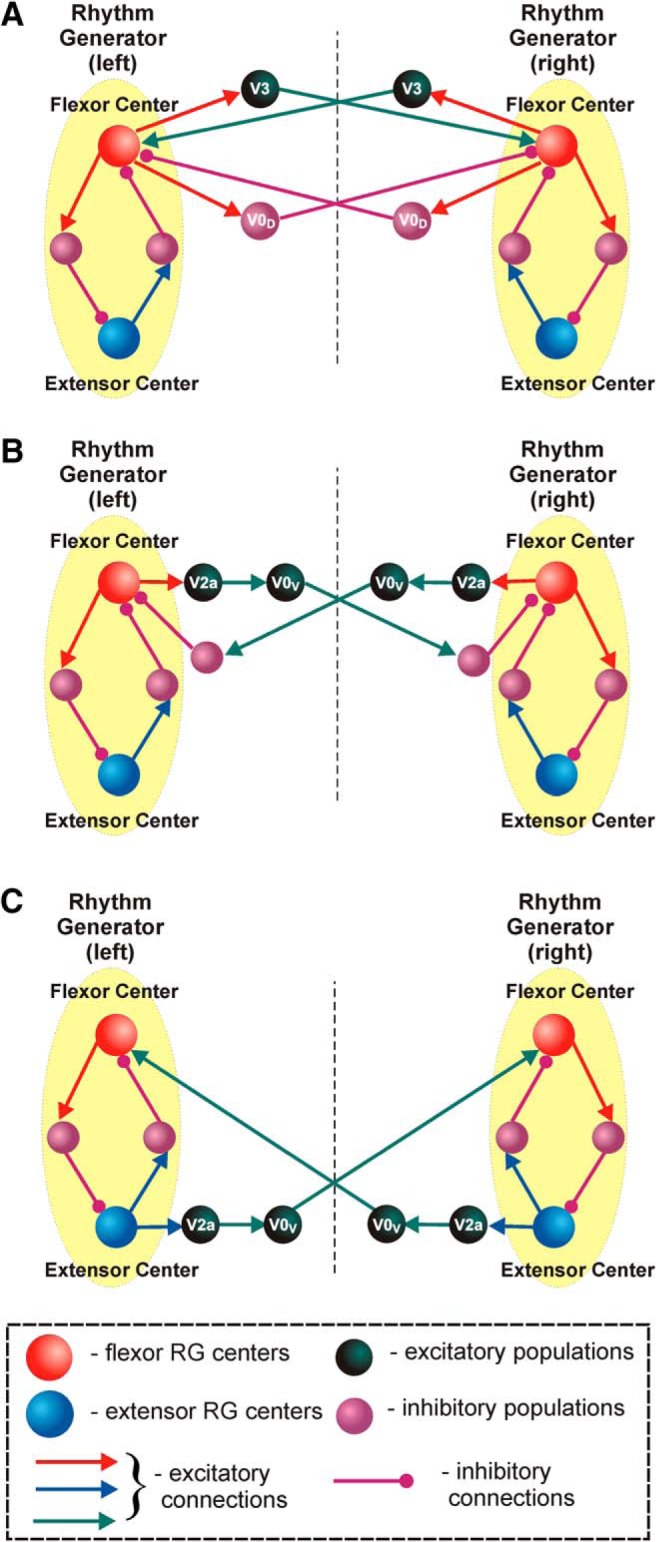
The proposed organization of the excitatory and inhibitory commissural pathways involved in left–right coordination of activity in the spinal cord. The RG centers and other neural populations are shown by spheres. Excitatory and inhibitory synaptic connections are represented by arrows and circles, respectively. ***A***, Organization of the excitatory (mediated by the V3 CINs) and inhibitory (mediated by the V0_D_ CINs) pathways. ***B***, ***C***, Two possible organizations of commissural pathways mediated by V2a interneurons and V0_V_ CINs.

As shown in Figure 5, *A* and *B*, a population of neurons with *I*_NaP_-dependent bursting properties and mutually excitatory synaptic interconnections can be silent, generate intrinsic bursting, or exhibit sustained (tonic) activity, depending on the average level of neuronal excitation (see also Butera et al., 1999; Smith et al., 2000; Rybak et al., 2003, 2004, 2014; Jasinski et al., 2013). In the model of Shevtsova et al. (2015), the rhythm-generating population was used for the simulation of both flexor and extensor centers. Therefore, under certain conditions, for example at a particular range of neuronal excitation, each flexor and extensor RG center can generate rhythmic bursting. However, it was assumed that, under the experimental conditions considered, only flexor centers operate in a bursting mode, whereas the extensor centers, if isolated, are tonically active. This was reproduced in the model of Shevtsova et al. (2015) by extensor RG centers with a higher level of excitation (Fig. 5*G*, yellow area) compared with the flexor RG centers (Fig. 5*G*, blue area). To provide alternating flexor–extensor activity, the flexor and extensor RG centers were connected reciprocally via inhibitory neural populations (Fig. 6*A*,*B*). Therefore, each extensor RG center also exhibited rhythmic bursting, but this was entirely due to the rhythmic inhibition by the corresponding ipsilateral flexor center. Such asymmetric operation of flexor and extensor RG centers allowed the model to reproduce the specific patterns of motor activity in the spinal cord observed during spontaneous, non-resetting burst deletions, showing that missing flexor bursts were always accompanied by a sustained ipsilateral extensor activity, whereas missing extensor bursts occurred without an effect on flexor bursting (Fig. 4A1, A2, see also Zhong et al., 2012).

## Left–right coordination of activity in the spinal cord: frequency-dependent role of V0_D_ and V0_V_ CINs

 The following types of CINs have been genetically identified in the spinal cord so far: the inhibitory V0_D_, the excitatory V0_V_, and the excitatory V3 CINs (Fig. 2). There are also dI6 neurons (Dyck et al., 2012), which are not considered here. The simplest connectivity schematic for the excitatory CIN pathways supporting left–right synchrony could be that in which these CINs (e.g., V3) would receive excitation from ipsilateral flexor centers and excite the contralateral flexor centers (Fig. 6*A*). In contrast, the V0 CINs, both the inhibitory V0_D_ and the excitatory V0_V_ types, support left–right alternation (Talpalar et al., 2013). The inhibitory V0_D_ CIN pathway could receive excitation from ipsilateral flexor centers and inhibit the contralateral flexor center (Fig. 6*A*).

The organization of the commissural pathways involving the excitatory V0_V_ CINs promoting left–right alternation is expected to be more complicated. As described above (see Spinal interneurons identified from developmental and genetic studies; Crone et al., 2008; Kiehn, 2011), inputs to V0_V_ CINs can be mediated by the ipsilaterally projected V2a neurons. Two possible organizations of V2a–V0_V_ pathways promoting left–right alternation can be considered. One potential configuration suggests that the V2a neurons from these pathways receive excitatory inputs from the ipsilateral flexor centers and excite V0_V_ CINs, whereas the V0_V_ CINs inhibit the contralateral flexor centers via some inhibitory interneurons (Kiehn, 2011, Talpalar et al., 2013; Shevtsova et al., 2015; Fig. 6*B*). The other possible configuration of V2a–V0_V_ pathways is based on excitatory inputs to the V2a neurons from the ipsilateral extensor centers and direct excitatory input from the corresponding V0_V_ CINs to the contralateral flexor centers (Shevtsova et al., 2015; Fig. 6*C*).

The frequency-dependent role of V0_D_ and V0_V_ neurons in left–right alternation of neural activity in the spinal cord was studied by Talpalar et al. (2013). They have shown that the ablation of both these types of CINs leads to left–right synchronized, hopping-like activity at all locomotor frequencies. Selective ablation of the inhibitory V0_D_ CINs disturbs left–right alternation at low frequencies, yet maintains alternation at high frequencies. In contrast, ablation of the excitatory V0_V_ CINs maintains alternation at low frequencies and switches to synchronized activity at high frequencies.

**Figure 7.**
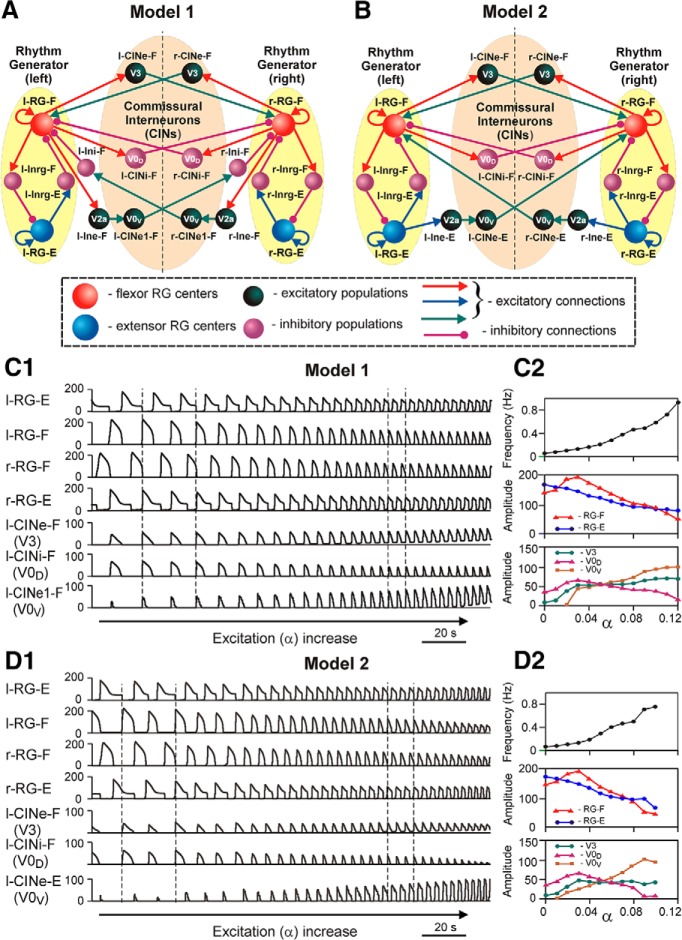
Modeling the coordination of left and right rhythm generators in the spinal cord via several parallel CIN pathways. ***A*** and ***B*** show schematics of Model 1 and Model 2, respectively. Neural populations are shown by spheres. Each RG population (representing the flexor or extensor centers) contained 200 neurons; all other populations consisted of 50 neurons each. All neurons were modeled in the Hodgkin–Huxley style. Excitatory and inhibitory synaptic connections are represented by arrows and circles, respectively. Prefix l- or r- in population name indicates left or right. The RG on each side of the cord includes flexor and extensor RG centers (RG-F and RG-E, respectively) interacting via the inhibitory Inrg-F and Inrg-E populations. Suffix F- or E- in population name indicates that the population is coactive with the flexor or extensor RG center, respectively. The left and right rhythm generators interact via CINs: CINe-F (V3), CINi-F (V0_D_), and CINe1-F (V0_V_) in Model 1 (***A***); and CINe-F (V3), CINi-F (V0_D_), and CINe-E (V0_V_) in Model 2 (***B***). The full mathematical description of the model can be found in the study by Shevtsova et al. 2015. Reproduced from Shevtsova et al. (2015), their Figure 1, with permission. ***C1*** and ***D1*** show the performances of Model 1 and Model 2, respectively, in response to a slow ramp increase of neuronal excitation. The activity of all four RG centers (left and right RG-F and RG-E populations) and left CIN populations are shown as average histograms of population activity [spikes/(neuron × s), bin = 100 ms] in response to a slow ramp increase of neuronal excitation α (α increased for 100 s). Note the maintenance of left–right and flexor–extensor alternation and acceleration of rhythmic activity in both models. The vertical dashed lines indicate the beginning of the left flexor phases at lower (the left lines) and higher (the right lines) values of α. Reproduced from Shevtsova et al. (2015), their Figure 4, with permission. ***C2*** and ***D2*** show changes of the key model characteristics in Model 1 and Model 2, respectively, in response to a slow ramp increase of neuronal excitation. In both panels, the top diagram shows changes in the frequency of oscillation; the middle diagram represents changes in the amplitude of the activity of l-RG-F (red) and l-RG-E (blue) centers; and the bottom diagram shows changes in the amplitude of activity of V3 (green), V0_D_ (purple), and V0_V_ (brown) CIN populations. Reproduced from Shevtsova et al. (2015), their Figure 5, with permission.

To reproduce and explain these findings using computational modeling, Shevtsova et al. (2015) developed two slightly different models, Model 1 and Model 2 (Fig. 7*A*,*B*). Each model contained left (l) and right (r) RGs consisting of flexor (RG-F) and extensor (RG-E) rhythm-generating centers reciprocally inhibiting each other via Inrg-E and Inrg-F, respectively. In both models, the inhibitory left (l-CINi-F) and right (r-CINi-F) CIN populations, simulating the V0_D_ CINs, mediate mutual inhibition between left and right flexor centers (Fig. 6*A*). Also, the excitatory left CIN flexor (l-CINe-F) and right CIN flexor (r-CINe-F) populations, simulating excitatory CINs, which are conditionally associated with the V3 neurons, mediate mutual excitation between these centers (Fig. 6*A*). In addition, both models have commissural pathways that include the sequentially connected V2a and V0_V_ populations. The only difference between the models is in the organization of the V2a–V0_V_ pathways. In Model 1, these pathways are organized as in Figure 6*B*, so that the left V2a flexor and right V2a flexor populations receive excitatory inputs from the ipsilateral flexor centers (l-RG-F and r-RG-F), and excite the corresponding V0_V_ extensor populations (l-CINe1-F and r-CINe1-F) which project to the contralateral populations of inhibitory interneurons that inhibit the contralateral flexor centers (Fig. 7*A*). In Model 2, these pathways are organized as in Figure 6*C*, so that the left and right V2a populations receive excitatory inputs from the ipsilateral extensor centers (l-RG-E and r-RG-E), and the corresponding V0_V_ populations (l-CINe-E and r-CINe-E) directly project to and excite the contralateral flexor centers (Fig. 7*B*). Separate consideration of these two models allowed independent investigation of each of the two V2a–V0_V_ pathways suggested. However, the authors have not excluded the possibility that both pathways are present in the real spinal cord network, which would result in a simple merging of both models into a single united model (Shevtsova et al., 2015).

To evaluate the performance of both models at different locomotor speeds, and the speed-dependent contribution of different CIN pathways and neuron types to the maintenance of left–right coordination, the locomotor frequency was progressively increased by increasing the parameter α that characterized an increase of average neuronal excitation (*Ē*_L_) in all neuron populations of the model (Fig. 5*G* and legend). Hence, the increase in α values simulated the increase in NMDA concentration that was used in the experiments in the study by Talpalar et al. (2013) to increase the frequency of locomotor oscillations.

The results of simulations using Model 1 and Model 2 are shown in Figure 7, *C1* and *D1*, respectively. For each model, the integrated activity of all four RG centers (l-RG-E, l-RG-F, r-RG-F, and r-RG-E) and all left CIN populations [l-CINe-F (V3), l-CINi-F (V0_D_), and l-CINe1-F (V0_V_) for Model 1; and l-CINe-F (V3), l-CINi-F (V0_D_), and l-CINe-E (V0_V_) for Model 2] are shown while the average neuronal excitation (α) was slowly increased. In each panel, the right CIN populations behaved similarly to the left ones and are not shown. In both models, increasing neuronal excitation resulted in the acceleration of rhythmic bursting with maintenance of left–right and flexor–extensor alternation throughout the whole range of α values.

The amplitude of activity of the RG-F centers in both models initially goes up and then decreases, resembling the amplitude changes in the isolated population (Fig. 5*D*) as well as in experimental recordings from the flexor (L2) ventral root (Fig. 5*F*, 7*C2*,*D2*, middle diagrams, red curves). Analysis of the activity of the three CIN populations in the models allows evaluation of the relative contribution of each population to left–right coordination at different levels of neuronal excitation. With an increase in α values, the amplitude of activity of each CIN population in both models is affected by two opposing processes. On one hand, the increase of intrinsic neuronal excitation per se should increase the amplitude of each CIN activity. On the other hand, the reduction of the amplitudes of activity of RG centers providing synaptic inputs to these populations should decrease the amplitude of CIN activity.

In the inhibitory V0_D_ CIN populations, the synaptic process dominates, due to the relatively low leakage conductance in these neurons in the models defining the low sensitivity of their membrane potentials to changes in α (imitating drug concentration). Therefore, the amplitude of their activity reduces similarly to that of the ipsilateral flexor RG centers that provide direct synaptic inputs to these populations (Fig. 7*C2*,*D2*, bottom diagrams, purple curves). As a consequence, V0_D_ populations strongly contribute to left–right alternation at low levels of excitation in the network when flexor RG output is maximal, whereas at high levels of excitation their role is significantly reduced as flexor RG amplitude decreases.

In the excitatory V3 CIN populations, the two opposing processes partly compensate for each other, and hence the amplitude of their activity (after an initial increase) remains relatively constant and does not depend much on α and oscillation frequency (Fig.7*C2*,*D2*, bottom diagrams, green curves).

The amplitudes of activity of the excitatory V0_V_ populations (CINe1-F in Model 1 and CINe-E in Model 2) are zero at small values of α and monotonically increase with α, despite the reduction in the amplitudes of activity in the corresponding RG centers (Fig. 7*C2*,*D2*, bottom diagrams, brown curves). The increase in V0_V_ activity is due to the strong activation and recruitment of V2a neurons mediating the RG inputs to V0_V_ CINs (Fig. 7*A*,*B*). Therefore, in both models, the excitatory V0_V_ CINs contribute more strongly to left–right alternation at higher levels of α. In Model 1, this contribution occurs because V0_V_ CINs inhibit the contralateral flexor centers via inhibitory interneurons (Fig. 7*A*). In Model 2, this contribution occurs because the V0_V_ CINs mediate excitation from each extensor center to the contralateral flexor center (Fig. 7*B*). Hence, in both of our models, both V2a and V0_V_ populations are critically involved in providing left–right alternation at high levels of excitation and high oscillation frequencies.

In the intact models, left–right alternation is maintained at all levels of excitation (Fig. 7*C1*,*D1*), because the synchronizing action of V3 CINs is overcome by alternating the actions of V0_D_ CINs at lower levels of excitation with those of V0_V_ CINs at higher levels of excitation. This is illustrated in the bottom diagrams of Figure 7, *C2* and *D2*, which show that the amplitude of V3 population activity (green) is less than the amplitude of V0_D_ population activity (purple) at low levels of α, and less than the amplitude of V0_V_ population activity (brown) at high levels of α.

**Figure 8.**
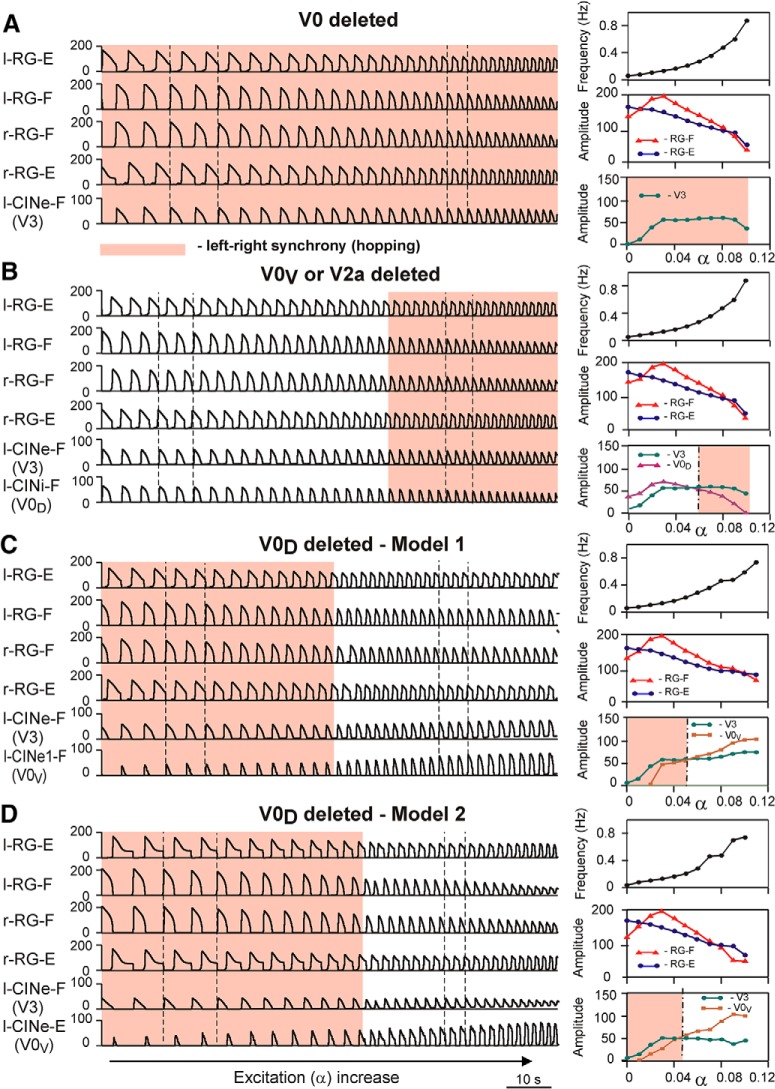
Frequency-dependent changes in left–right coordination of activity following selective removal of particular CIN pathways. ***A***, Performance of both models after removal of both V0 (V0_V_ and V0_D_) CIN populations. ***B***, Performance of both models after selective removal of V0_V_ CIN or V2a populations. ***C***, ***D***, Performance of Model 1 and Model 2, respectively, after selective removal of V0_D_ CIN populations. The left column in each panel shows the activity of all centers and the remaining (left) CIN populations. In all panels, α was increased for 200 s. The vertical dashed lines indicate the beginning of the left flexor phases at lower and higher values of α (and oscillation frequency). The right column in each panel shows changes of the key model characteristics in response to a slow ramp increase of neuronal excitation: the top diagram shows changes in the frequency of oscillation; the middle diagram represents changes in the amplitude of activity of l-RG-F (red) and l-RG-E (blue) centers; and the bottom diagram shows changes in the amplitude of activity of V3 (green), V0_D_ (purple), and V0_V_ (brown) CIN populations. The dash-dotted vertical lines in right panels indicate transitions between left–right alternation and left–right synchronization. The regions of left–right synchronized activity are highlighted by pink rectangles. Modified from Shevtsova et al. (2015), their Figures 7 and 8, with permission.

Let us now consider how these models behave following a removal of all V0 CIN populations, only the V0_V_ or V2a populations, or only the V0_D_ populations (Fig. 8).

When both types of V0 populations are deleted (Fig. 8*A*), the only remaining commissural pathways in both models are mediated by the excitatory V3 populations (Fig. 8*A*, right column, bottom diagram). In this case, V3 CINs synchronize oscillations of the left and right flexor RG populations at all levels of neuronal excitation and any locomotor frequency, resulting in a “hopping” pattern.

If only V2a-V0_V_-mediated (Fig. 8*B*) or only V0_D_-mediated (Fig. 8*C*,*D*) pathways are selectively deleted, the model behavior depends on the interplay between the V3-mediated left–right synchronization and the remaining V0 populations that support left–right alternation. Figure 8*B* shows that when either the V0_V_ or the V2a populations are deleted from either model, the left and right flexor RG centers, as well as the left and right extensor RG centers, exhibit alternating activity at lower values of α (low locomotor speeds) and synchronous activity at higher values of α (higher locomotor speed). The bottom diagram in the right column of Figure 8*B* shows that the amplitude of activity of the remaining inhibitory V0_D_ CIN populations decreases with increasing α. In contrast, the amplitude of the excitatory V3 CIN populations (l-CINe-F and r-CINe-F) after an initial increase remains relatively constant. As a result, at some value of excitation, the activity of the V3 populations becomes stronger than the activity of the V0_D_ populations, leading to the cross-cord synchronization of activity of the left and right flexor and of the left and right extensor RG centers. In summary, removal of V0_V_ CIN or V2a populations results in left–right alternation of neuronal activity at lower levels of neuronal excitation (low locomotor speeds) and left–right synchronization at higher levels of neuronal excitation (higher locomotor speeds).

Figure 8, *C* and *D*, demonstrates the results of simulations using Model 1 and Model 2, respectively, after removal of V0_D_ populations. In both models, left and right homonymous RG centers exhibit synchronous activity at lower values of α and switch to alternating activity at higher values of α. In both models, the amplitude of activity of the V0_V_ CIN populations increases with increasing α (Fig. 8*C*,*D*, right column, bottom diagram in both) because of the dramatic increase in the activity of V2a populations exciting these V0_V_ CIN populations (Fig. 7*A*,*B*). At the same time, the amplitude of the V3 CIN populations (l-CINe-F and r-CINe-F) remains relatively constant. Eventually, the amplitude of activity of the V0_V_ CIN populations promoting alternation becomes stronger than the amplitude of V3 CIN populations promoting synchronization. Therefore, in both our models, the removal of V0_D_ CIN populations leads to left–right synchronization of neuronal activity at lower levels of neuronal excitation (low locomotor speeds) and left–right alternation at higher levels of excitation (higher locomotor speeds).

The simulation results of Shevtsova et al. (2015) described above closely reproduce the experimental data of Talpalar et al. (2013). They support the earlier suggestion that left–right alternation is provided by dual commissural pathways involving the inhibitory V0_D_ and excitatory V0_V_ CINs, and that the contribution of the V0_D_ pathway to left–right alternation is dominant at low frequencies and reduces as locomotor frequency increases, whereas the contribution of the V2a–V0_V_ pathway is weak at low frequencies but is enhanced as frequency increases. The models suggest that these important features may be based on the following: (1) a relatively weak dependence of excitation of the V0 CINs on neuroactive drug concentration, which leads to a net reduction of V0_D_ activity due to the reduction in the amplitude of rhythm-generating activity when the frequency increases; and (2) a strong dependence of V2a neuron activity and recruitment on the neuroactive drug concentration (Crone et al., 2009; Zhong et al., 2011); these neurons mediate input to V0_V_ CINs, hence providing a net increase of their activity with frequency. A more detailed explanation of this suggestion can be found in the study by Shevtsova et al. (2015).

The two models described in this study show very similar behavior. In both models, the V0_D_ populations receive input from the flexor RG center and inhibit the contralateral flexor RG center. Based on this, our models predict that V0_D_ CINs should exhibit flexor-related rhythmic activity, which can be tested in future experiments. The two models differ by the organization of V2a–V0_V_ pathways and the phase of activity of these neurons. Specifically, the V2a–V0_V_ neurons may (1) coordinate left and right flexor centers via inhibitory interneurons as predicted by Model 1, (2) mediate connections from each extensor center to the contralateral flexor center as predicted by Model 2, or (3) include both of these pathways (Shevtsova et al., 2015). The choice between these models can be made in the future based on recording from V0_V_ neurons, and may depend on whether their activity is mostly in phase with the flexors (confirming Model 1), with extensors (confirming Model 2), or with both types (confirming option 3). It is certainly possible that the two V2a–V0_V_ pathways are both present in the spinal network, because V2a neurons with both flexor- and extensor-related rhythmic activity have been previously described (Dougherty and Kiehn, 2010; Zhong et al., 2010). Moreover, other, possibly more complicated constructions of commissural pathways, mediated by V0_D_, V0_V_, V3 CINs, their unknown subtypes, or other CINs, could be considered and represent a subject of future studies.

## Axon guidance and left–right coordination: role of *Netrin-1*, *DCC*, and *EphA4*

 As described above, the coordination of left–right activity in the spinal cord is provided by multiple commissural pathways mediated by different CIN types, whose axons cross midline and affect neurons on the contralateral side of the cord. During spinal cord development, this process is directed by axon-guiding molecules such as *Netrin-1* that bind to and activate the axonal receptor *DCC* (Kennedy et al., 1994; Kaprielian et al., 2001; Rabe et al., 2009; Rabe Bernhardt et al., 2012; Vallstedt and Kullander, 2013). The other axon-guidance molecule involved in the development of connectivity across the midline of the spinal cord is *EphA4* (Dottori et al., 1998; Kullander et al., 2003).

Modeling of the spinal cord reorganizations following the genetic removal of axon-guidance molecules, such as *Netrin-1*, *DCC*, and *EphA4* was performed by Rybak et al. (2013) using a reduced model of left and right flexor rhythm generators interacting via unnamed inhibitory and excitatory CINs involved in left–right coordination of activity. This model was then used by Shevtsova et al. (2015) as a basis for developing the more elaborated and complicated models that explicitly incorporated genetically identified V0_D_, V0_V_, and V3 CINs (Models 1 and 2 described in the previous section). Similar to the basic model of Rybak et al. (2013), these later models can simulate spinal circuit reorganizations following genetic removal of the above axon-guiding molecules and reproduce the corresponding experimental data (N. A. Shevtsova and I. A. Rybak, unpublished results). Below, we interpret the above circuit reorganizations using the network architecture of Model 1 (Fig. 7*A*), although the slightly different schematic of Model 2 could be used as well.

**Figure 9.**
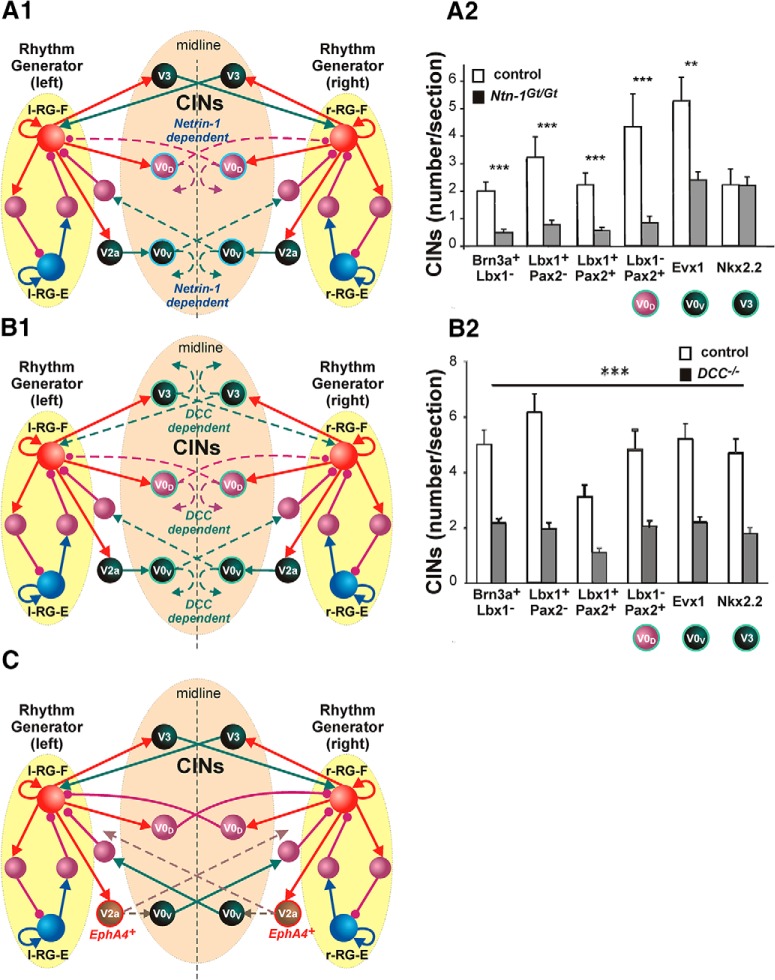
Potential changes in left–right coordination in models of mutants lacking particular axon-guiding molecules *Netrin-1*, *DCC*, or *EphA4*. ***A1***, Model of circuits in *Netrin-1* KO mice. The majority of axons of V0_D_ and V0_V_ CINs do not cross the midline (dashed lines), hence reducing mutual inhibition between left and right RGs. ***A2***, Quantification of transcription factor phenotype in different traced CINs that shows significant differences in affected V0_D_ and V0_V_ CIN (but not V3) populations in *Netrin-1* KO mice. Modified from Rabe et al. (2009), their Figure 5L, with permission. ***B1***, Model of circuits in *DCC* KO mice: significant portions of axons of V0_D_ and V0_V_, and V3 CINs do not cross the midline (dashed lines), hence reducing both mutual inhibition and mutual excitation between left and right RGs. ***B2***, Quantification of transcription factor phenotype in different traced CINs that shows significant differences in affected V0_D_, V0_V_, and V3 CIN populations in *DCC* KO mice. Modified from Rabe Bernhardt et al. (2012), their Figure 4I, with permission. ***C***, Model of circuits in *EphA4* KO mice: many axons of EphA4^+^ V2a populations do not activate V0_V_ CINs, hence reducing mutual inhibition between left and right RGs. In ***A1***, ***B1***, and ***C***, both the right connections with reduced numbers of axons and the redirected axons in the corresponding mutant circuits are shown by dashed lines.

*Netrin-1* is found in the floor plate and neuroepithelial cells of the ventral region of the spinal cord and is involved in attracting CIN axons and directing them to the contralateral side of the cord. Figure 9*A2* (modified from Rabe et al., 2009, with permission) shows that the loss of *Netrin-1* in the knockout (KO) mice significantly reduces the number of axons crossing the midline for V0_D_ (Lbx1^−^ Pax2^+^) and V0_V_ (Lbx1^−^ Pax2^+^) CINs without changing the number of V3 (Nkx2.2) axons crossing the midline. This reduction in the number of midline-crossing axons is shown in the model schematic in Figure 9*A1* by dashed lines, illustrating that, although some V0_D_ and V0_V_ connections still go to the contralateral side (as in the intact model), some of their connections stay on the ipsilateral side (Fig. 9*A1*, curved dashed arrows). These changes in commissural interactions should reduce mutual inhibition between left and right RGs mediated by V0_D_ and V0_V_ CINs, and allow domination of the V3-mediated pathways synchronizing the activity of left and right RGs leading to a hopping pattern, similar to that in the fully V0 removed case (Fig. 8*A*). This shift in the left–right commissural interactions to domination by left–right excitation provides a clear explanation of the experimental data on left–right synchronous activity and hopping gate in *Netrin-1* KO mice (Rabe et al., 2009; Vallstedt and Kullander, 2013).

In *DCC* KO mice, the number of midline-crossing axons becomes reduced for both the CIN types supporting left–right alternation: V0_D_ (Lbx1^−^ Pax2^+^) and V0_V_ (Lbx1^−^ Pax2^+^), and the V3 (Nkx2.2) CINs promoting left–right synchrony [Fig. 9B2 (reprinted with permission from Rabe Bernhardt et al., 2012)]. This is shown in the model schematic in Figure 9*B1* by the dashed lines, illustrating that some of the V0_D_, V0_V_ and V3 connections do not cross midline and stay ipsilaterally. These should strongly reduce all left–right interactions, leading to an uncoordinated left–right activity (Rybak et al., 2013). This reduction of commissural interactions that is responsible for the coordination of left and right activities provides an explanation of the experimental data on uncoordinated left–right activity in *DCC* KO mice (Rabe Bernhardt et al., 2012; Vallstedt and Kullander, 2013).

*EphA4* ^+^ neurons are repelled by *ephrin-B3* expressed at the midline of the spinal cord. In *EphA4* KO mice, the regular left–right alternating walking pattern is replaced with a rabbit-like left–right synchronized hopping gait (Dottori et al., 1998; Kullander et al., 2003; Akay et al., 2006). It has been initially suggested that in the *EphA4* KO mice, the normal left–right alternating activity, which usually results from crossed net inhibition, is overridden by the abnormal crossed excitatory interactions (Butt et al., 2005; Kiehn, 2006; Restrepo et al., 2009, Rybak et al., 2013). Studies in spinal cords isolated from *EphA4* KO mice have confirmed that switching to the synchronized hopping pattern is accompanied by, and supposedly results from, an abnormal midline crossing of axons of some excitatory neurons that normally stay ipsilateral to the spinal cord (Restrepo et al., 2009). However, based on the information that V2a neurons, projecting to V0_V_ CINs (Figs. 7*A*,*B*, 9*C*), are *EphA4* ^+^ (Lundfald et al., 2007), it is reasonable to suggest that switching to left–right synchronous hopping-like activity in *EphA4* KO mice may result from a reduction of the number of V2a axons projecting to the ipsilateral V0_V_ CINs, because some of these axons—due to the lack of *EphA4* molecules—cross the midline and project to the other side of the cord (Borgius et al., 2014). This redirection of V2a axons in *EphA4* KO mice is illustrated in Figure 9*C* by the double dashed arrows out of V2a neurons, one of which crosses the midline. This redirection of V2a axons reduces the inhibitory commissural interactions mediated by the V2a–V0_V_ pathways that secure left–right alternation in the intact mice and allows their action to be overcome by the V3 pathways, promoting left–right synchronizing, leading to the left–right synchronized hopping behavior.

## Flexor–extensor alternation: role of V1 and V2b interneurons

 Reciprocal activation of flexor and extensor muscles represents the fundamental mechanism involved in any motor behaviors, including locomotion. The spinal cord circuits providing flexor–extensor alternation have been studied recently by Zhang et al. (2014; see also Goulding et al., 2014). They have shown that the alternating flexor–extensor activity during locomotion depends on two classes of ventrally located inhibitory neurons: V1 and V2b. Abrogating V1 and V2b interneuron-derived neurotransmission in the isolated spinal cord results in a synchronous pattern of flexor-related (L2) and extensor-related (L5) locomotor activity. Mice lacking V1 and V2b inhibition are unable to move their limb joints and display marked deficits in limb-driven reflex movements.

Note that the population of V1 interneurons is composed of multiple functionally different cell types, including Ia inhibitory interneurons and Renshaw cells mediating different forms of inhibition at the level of motoneurons (Goulding, 2009). Here, we use the term “V1 neurons” only for the subpopulation of V1 neurons that are potentially involved in flexor–extensor and left–right interactions at the CPG (not motoneuron) level (i.e., for V1 cells not representing Ia or Renshaw cells).

The recent study by Zhang et al. (2014) has shown that, although both V1 and V2b interneurons are involved in flexor–extensor alternation, their functional roles are different, and their selective removal from the intact cord and isolated hemicord produces different effects on locomotor pattern. The major findings of the study by Zhang et al. (2014) are summarized as follows: (1) removal of V1 neurons results in a significant reduction in the frequency of drug-induced locomotor activity in the isolated intact cord (two to three times and more), which is similar to the frequency reduction observed after cord hemisection, and a lack of V1 neurons has no observed effects on the hemicord rhythmic activity; and (2) both interneuron types, V1 and V2b, secure flexor–extensor alternation in the intact cord, so that only removal/silencing of both types leads to flexor–extensor synchrony. In contrast, flexor–extensor alternation in the isolated hemicord is entirely supported by V2b neurons, whose silencing results in flexor–extensor synchrony.

**Figure 10.**
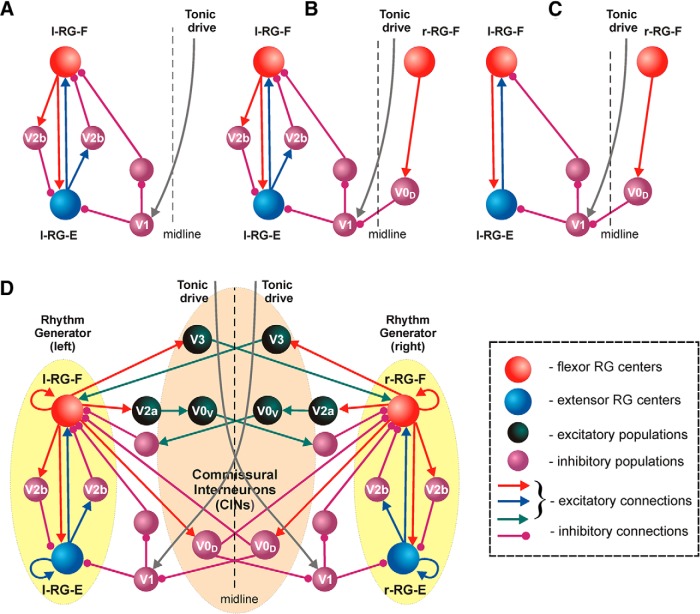
Role of V1 and V2b interneurons in flexor–extensor alternation. ***A***, ***B***, V2b neurons mediate mutual inhibition between flexor and extensor half-centers on each side of the cord. V1 neurons (involved in interactions between rhythm-generating centers) receive excitation from the contralateral side of the cord. This excitatory input should include tonic excitatory drive. These V1 neurons provide an additional inhibition of the ipsilateral extensor center and may disinhibit the ipsilateral flexor half-center. ***B***, The activity of the above V1 cells is negatively modulated (reduced) in phase with the contralateral flexor activity via inhibition from the contralateral V0_D_ CINs. ***C***, The above modulation of V1 neuron activity allows them to secure flexor–extensor alternation after removing V2b neurons in the intact cord. ***D***, The full schematic of bilaterally interacting rhythm generators in the spinal card with incorporated V1 and V2b circuits.

Based on the data from the study by Zhang et al. (2014), the following features of organization of V2b and V1 circuits involved in flexor–extensor alternation and locomotor frequency control can be hypothesized:

(1) The V2b neurons mediate mutual inhibition between flexor and extensor half-centers on each side of the cord (Fig. 10*A*,*B*).

(2) The V1 neurons (involved in interactions between rhythm-generating centers) receive excitation only from the contralateral side of the cord, and hence they should not be active and operate in isolated hemicords (i.e. after hemisection; Fig. 10*A*,*B*).

(3) The excitatory input to V1 neurons from the contralateral side of the cord includes tonic excitatory drive (Fig. 10*A–C*) that maintains sustained activity of V1 neurons in the intact cord. This tonic drive can be provided by separate types of commissural interneurons that are activated by drugs in this preparation, but they are normally activated by input from the mesencephalic and cerebellar locomotor regions and send their axons to the contralateral side of the spinal cord (Jankowska and Noga, 1990; Matsuyama and Mori, 1998; Bannatyne et al., 2003; Jankowska et al., 2003, 2005).

(4) When active (in the intact cord), these V1 neurons provide an additional inhibition of the ipsilateral extensor half-center and may disinhibit the ipsilateral flexor half-center (Fig. 10*A–C*), hence allowing the generation of high locomotor frequencies in the intact cord. Therefore, removal/inactivation of these neurons in the intact cord or hemisection should lead to slowing the locomotor frequency. These V1 neurons may also serve as targets for the supraspinal control of locomotor speed in the intact system (Gosgnach et al., 2006; Zhang et al., 2014).

(5) The activity of the V1 cells is negatively modulated (reduced) in phase with the contralateral flexor activity, for example via inhibition from the contralateral V0_D_ CINs (Fig. 10*B*,*C*). This rhythmic reduction of V1 inhibition in phase with contralateral flexor activity allows these neurons to secure flexor–extensor alternation after removing V2b neurons in the intact cord (Fig 10*C*).

Figure 10*D* shows an updated schematic of the model of Shevtsova et al., 2015; Fig. 7*A*, Model 1, that incorporates the V1 and V2b circuits hypothesized above (a similar modification can be applied for Model 2). In this architecture, a removal of V1 population in the intact cord or a hemisection (which removes the contralateral excitatory drive to V1 populations) would lead to a disinhibition of extensor centers and additional inhibition of flexor centers (via a disinhibition of interposed inhibitory interneurons), leading to slowing down the locomotor oscillations. This would be consistent with point 1 above. Also in this architecture, both V2b (explicitly) and V1 populations (due to inhibitory modulation of its activity by the contralateral V0_D_ population) would secure flexor–extensor alternation in the intact cord, but only V2a populations would be able to do this after hemisection silencing V1 neurons. This would provide point 2 above.

It is expected that this united architecture will be able both to maintain all the features of the locomotor network described above and to reproduce the major findings of the study by Zhang et al. (2014) concerning the role of V1 and V2b neurons for flexor–extensor alternation and frequency control.

## Conclusions

 Our analysis has focused on using some logical and modeling approaches that allowed us to explicitly incorporate several known genetically identified neuron types into a hypothesized network architecture, representing a connectome of spinal circuits that includes the left and right CPGs interacting via several distinct CIN types (V0_D_, V0_V_, V3) and ipsilaterally projecting neurons (V1, V2a, V2b). We realize that other, currently unknown classes of spinal interneurons with different properties and connectivity patterns can be found in the future, and that some of the currently identified classes can be subdivided into functionally different subclasses. Nevertheless, we believe that the network architectures considered here could serve as a basis for future, more elaborated models of the spinal circuits. These network architectures propose plausible explanations for multiple experimental data obtained in different laboratories under different experimental conditions. The predictions of this study include the following: (1) flexor–extensor asymmetry in the organization of rhythm-generating locomotor network; (2) organization and frequency-dependent interactions of commissural pathways supporting left–right alternation (V0_D_, V2a/V0_V_, V3) and synchronization (V3) of spinal cord activity, which are potentially involved in locomotor gait control; and (3) organization of circuits of ipsilaterally projecting V1 and V2b neurons providing flexor–extensor alteration and locomotor frequency control. These predictions provide important insights into the organization of spinal locomotor CPGs and neural control of locomotion, and await experimental testing and validation.

## Synthesis

The decision was a result of the Reviewing Editor Douglas Bayliss and the peer reviewers coming together and discussing their recommendations until a consensus was reached. A fact-based synthesis statement explaining their decision and outlining what is needed to prepare a revision is listed below. The following reviewers agreed to reveal their identity: Patrick Whelan, Ronald Calabrese

Both reviewers provide appreciative comments regarding the timeliness and content of this review, which attempts to summarize the current state of understanding of the specific neuronal populations within the spinal cord that comprise the locomotor CPG and uses modeling data to propose network configurations that may predict/explain experimental observations of the spinal locomotor network. The major concern with the review manuscript in its current form is the presentation, in later sections, of new modeling results that have not been independently peer reviewed. You may choose to deal with this issue in either of two ways: 1) omit the new modeling results and keep only those aspects of the text that can be supported by published data; or 2) hold the manuscript until you have had the opportunity to get the new modeling data reviewed and published (or accepted for publication) in an original research paper. I would note that there was a sense that the remaining material would likely be of sufficient interest to warrant publication if the decision was to omit and revise.

Please note a number of additional comments regarding the text that you should consider in preparing a revised manuscript (see the reviews below and a markup of the manuscript PDF file, which will be made available to you at the journal submission website.)

------

Reviewer #1

General Comments

This manuscript reviews an important topic involving the modelling of genetically defined circuits within the spinal cord. About a decade ago data began to be gathered regarding the functional role of transcription-factor identified interneurons in the control of locomotion. With the advent of optogenetics there has been renewed interest in the selective activation of spinal circuits.

Work has now matured to a point where modelling of the circuit can start to provide meaningful predictive data. The authors provide an review of recent modelling data compiled from their recent publications in 2015 - specifically the Journal of Physiology article. This provides a review of these data with a different spin.

Major Comments

1. It is interesting and perhaps important that the two different model configurations 'model I' and 'model II' behave similarly even when elements of the circuits are 'knocked out'. There are possibly other configurations which also emulate the findings. The existence of other configurations should be discussed.

2. I would have appreciated a franker discussion of the challenges of modeling a complex vertebrate system and assumptions made. This does not have to be posed as a negative. I think it is important to also acknowledge that the classes of interneurons are very likely to be subdivided over time. This of course represents progress. But it needs to be considered in the text.

Minor Comments

1. The authors consider the network in one state only. This is fine and indeed required to begin to pool data. That said it should be pointed out that the network configurations obtained are likely one of many.

2. A general issue is the availability of models to readers. I don't know about the policy of elife but it would be wonderful if the model code could be made available so that interested readers can use it.

3. Introduction - line 1 eliminate 'special'.

4. Delete/reword 'mutually consistent explanations'.

5. In introduction perhaps useful to discuss predictive value of models - one or two sentences. Perhaps discuss in context of simpler vertebrate networks - lamprey.

6. The sentence containing 'are too weak' is a little vague since 'strong' and 'weak' are not defined. Also sufficient to 'define, change and coordinate phase' does not sound weak to me. They could have more efficacy onto PF rather than RF circuits for example.

7. Page 8. 'is also maintaining' reword.

8. May want to provide a bit more detail regarding V3's - i.e. participate in maintaining a stable, symmetrical rhythm.

9. Page 9. 'Or they are spread thinly' change thinly to sparsely.

10. Page 9. Related to 9 above could discuss the concept that the neurons the RF neurons form part of a distributed network of multiple classes of neurons. This would create a redundancy that could explain why knocking out a class has a small effect.

11. Page 9/10. The case for Hb9 cells is tricky at the moment due to issues in selectively activating and inactivating this class of cells. Also evidence suggests that their activity do not precede burst initiation but may contribute to maintenance. May want to include this.

12. I don't know what 'quasi-symmetrical' is supposed to mean. And I don't think it likely that Graham-Brown would have described them as such.

13. Page 12. 'duration of flexor bursts'. The fact that extensor bursts duration changes more relative to flexor bursts has also been well described in adult walking preparations - e.g. the cat.

14. Page 13. 'But the rhythmicity of extensor RG circuits....may depend on type of experimental preparation used'. I agree with this statement and understand the conditional aspect but it could equally be a caveat for all proposed circuits.

15. Page 14. 'However, it was assumed that,....Extensors, if isolated, are tonically active' Is this a reasonable assumption? Work by Kiehn, also Lev-Tov show rhythmogenic capacity in isolated caudal lumbar segments.

16. Page 25. Interesting to see that the authors are suggesting that V1 interneurons present a heterogeneous pool and the authors are considering only a sub-population. This should be moved up as it is an important clarification.

Reviewer #2

General Comments

The authors present an interesting review of the current state of understanding of the mammalian spinal locomotor CPG. This review depends heavily on modeling results, most of which are published, to explain detailed experimental observations of the spinal locomotor network. The writing is in general clear and although the manuscript is challenging to read because of the complicated nature of the neuronal networks involved, the authors do a good job of helping the reader and the manuscript is very informative. The Figures although very complicated and difficult to assimilate are ultimately helpful and informative. I will confess that I do not work on spinal cord but I am an expert on CPGs and am very interested in spinal CPGs so I really wanted to review this paper so I could catch up to the field. I hope the authors are aiming people like me, so they will not mind me saying that they need to do more work to make the difficult circuitry accessible. Many suggested minor edits are peppered throughout the text on the pdf. Also, do all you can to minimize the alphabet soup. In addition to these more minor concerns I have one major concern.

Major Concern:

1. The last two sections of the review are both difficult to follow (can be helped by rewriting to amplify explanations) and bordering on stretching this paper into the realm of a scientific report. Especially the last section seems to present new not previously peer reviewed simulation results and the penultimate section presents reasoning from existing models that is non-quantitative but also borders on novel modeling. If I knew the authors of the paper then maybe I could be reassured that they are appropriately using the model of Shevtsova et al. 2015 and/or Molkov et al. 2015, but I cannot know that, and even if I did this is stretching what a review should be. There is no description of the methods, e.g. I leave it to the editor to determine if such new results can be presented in a review. If they are the authors should at least amplify the description in the final two sections of the review so that a more general reader like myself can follow more easily.
